# Hepatitis C virus infection status and associated factors among a multi-site sample of people who used illicit drugs in the Amazon region

**DOI:** 10.1186/s12879-019-4270-2

**Published:** 2019-07-17

**Authors:** Aldemir B. Oliveira-Filho, Francisco Junior A. Santos, Fabricio Quaresma Silva, Nairis Costa Raiol, Camila Carla S. Costa, Juliana Nadia F. Piauiense, Luisa Caricio Martins, Yasmin Maria N. Cardoso, Jeruza Ferraz F. Di Miceli, Rafael Lima Resque, Gláucia C. Silva-Oliveira, Luiz Marcelo L. Pinheiro, Luiz Fernando A. Machado, João Renato R. Pinho, José Alexandre R. Lemos, Emil Kupek, Benedikt Fischer

**Affiliations:** 1 Laboratório de Células e Patógenos, Grupo de Estudo e Pesquisa em Populações Vulneráveis (GEPPOV), Instituto de Estudos Costeiros, Campus de Bragança, Universidade Federal do Pará, Alameda Leandro Ribeiro, s/n. Aldeia, Bragança, PA Brazil; 2 Laboratório de Patologia Clínica de Doenças Tropicais, Núcleo de Medicina Tropical, Universidade Federal do Pará, Belém, PA Brazil; 3 Laboratório de Toxicologia e Química Farmacêutica, Centro de Ciências Biológicas e da Saúde, Universidade Federal do Amapá, Macapá, AP Brazil; 4 Faculdade de Ciências Biológicas, Campus do Marajó – Soure, Universidade Federal do Pará, Soure, PA Brazil; 5 Laboratório de Virologia, Instituto de Ciências Biológicas, Universidade Federal do Pará, Belém, PA Brazil; 6 Laboratório de Gastroenterologia e Hepatologia, Instituto de Medicina Tropical, Universidade de São Paulo, São Paulo, SP Brazil; 7 Programa de Pós-Graduação Profissional em Análises Clínicas, Instituto de Ciências Biológicas, Universidade Federal do Pará, Belém, PA Brazil; 8 Departamento de Saúde Pública, Universidade Federal de Santa Catarina, Florianópolis, SC Brazil; 9 Institute for Mental Health Policy Research, Centre for Addiction and Mental Health (CAMH), Toronto, Canada; 10 Department of Psychiatry, University of Toronto, Toronto, Canada; 11 Faculty of Medical and Health Sciences, University of Auckland, Grafton, AK New Zealand

**Keywords:** HCV, Epidemiology, Spontaneous clearance, People who used illicit drugs, Brazil, Amazon

## Abstract

**Background:**

Elevated rates of Hepatitis C Virus (HCV) infection have been reported in epidemiological studies with people who used illicit drugs (PWUIDs) in different Brazilian regions. In Brazil’s Amazon region, studies have already identified the common use of illicit drugs among adolescents and the high prevalence of HCV infections among PWUIDs. However, all studies done with PWUIDs were conducted with small samples and within limited geographic coverage. This study determined the prevalence and risk factors for HCV infection in PWUIDs in the Amazon region, northern Brazil, as well as estimating the prevalence and factors associated with the HCV spontaneous clearance (HSC).

**Methods:**

This cross-sectional study accessed 1666 PWUIDs from multiple municipalities of the Amazon region. Socio-demographic, economic, drug use and health-related information were collected through interviews. Blood samples collected were tested for the presence of anti-HCV antibodies and RNA-HCV. HCV genotypes were identified by real-time polymerase chain reaction (PCR). Logistic regressions were run to identify factors independently associated with HCV infection status and HSC.

**Results:**

In total, 577 (34.6%) featured HCV antibodies, of which 384 (23.1%) had active HCV infection and 193 (11.6%) indicated HSC. Genotypes 1 (80.2%) and 3 (18.8%) were detected. HCV infection status was associated with the length of illicit drug use history, factors related to parenteral and sexual transmission, and factors of socio-economic marginalization leading to potential risk activities for HCV. HSC was associated with the ethnic (including indigenous) background of participants.

**Conclusions:**

High levels of HCV infection were detected in PWUIDs. Genotype 1 was predominant. Intense use of illicit drugs, unprotected sexual intercourse, high number of sexual partners and social marginalization were associated with all HCV infection. HSC was associated with origin (Amazonian-born) and non-white (e.g., Black or Indigenous) of PWUIDs. These findings emphasize the need for improve HCV prevention and control services and care for PWUIDs in the Brazilian Amazon region.

**Electronic supplementary material:**

The online version of this article (10.1186/s12879-019-4270-2) contains supplementary material, which is available to authorized users.

## Background

The hepatitis C virus (HCV) infection causes acute and chronic liver disease and may lead to cirrhosis, liver failure or hepatocellular carcinoma [1]. Globally, it is estimated that there are approximately 100 million persons with serological evidence of active or non-active HCV infection, and that HCV causes about 700,000 deaths each year [2]. Most cases of acute infection are asymptomatic and remain undiagnosed [3]. Currently highly effective pharmacotherapeutic (direct-acting antiviral) treatment options for HCV are now available [4]. Spontaneous clearance of HCV can occur within 12 months of infection in 15–45% of infected individuals, in the absence of treatment [5]. HCV infection does not result in long-term immunity and reinfection after effective treatment or clearance has been reported [6]. In epidemiological scenarios with high prevalence of HCV, multiple exposures may result in infections with HCV of more than one distinct genotype in the same person [7].

Illicit drug use – especially by injection and sharing non-sterile or shared injection equipment – is a main risk factor for transmission of HCV [8]. Many people who used illicit drugs (PWUIDs) infected with HCV are asymptomatic and/or unaware of their infection, and the continuation of risk activities that contribute to further transmission and an increase in the incidence of HCV [9]. Estimates indicate that there are about 16 million individuals who inject drugs worldwide, and unsafe use or sharing of drug paraphernalia (mainly syringes/needles used for drug injecting) is the main agent of virus transmission among users [10, 11]. As many as 10 million people who inject drugs may be HCV-infected worldwide, reflecting the strong association of these risk activities with the easy spread of the virus [10, 12].

Brazil is one of the emerging economies where the use of mostly stimulant drugs, such as cocaine – used intranasally (powder) or smoked (crack-cocaine and its related forms - merla or oxi) – has increased significantly in the last two decades [13–15]. Although uncommon, the use of injectable cocaine has been documented also [13, 16, 17]. There are several reasons for the high rate of cocaine-products used in Brazil: geographic proximity and un-secured borders with the world’s largest cocaine producers (Peru, Colombia and Bolivia), a young population combined with increases in wealth in the last decade, and the extensive availability combined with the cheap price of cocaine in Brazil (one crack ‘stone’ selling for three Reais, equivalent to US$ 1) [15, 18].

Elevated rates of HCV infection have been reported in epidemiological studies with PWUIDs in different Brazilian regions (2 to 29%) [19–23]. In the Amazon region (Northern Brazil), studies with PWUIDs have indicated a higher prevalence of HCV infection (28 to 37%), highlighting the elevated potential for the acquisition and transmission of HCV in this risk population [16, 17, 24, 25]. HCV genotype 1 is predominant among HCV-infected PWUIDs. Several risk factors for HCV infections have been reported: age (≥ 35 years), tattoos, domestic re-use of needles or syringes for medical procedures, injecting drug use, sharing of drug paraphernalia, daily and lengthy drug use histories (> 3 years). However, all studies done with PWUIDs were conducted with small samples and with limited geographic coverage [16, 17, 24, 25].

The vast Amazon region in Northern Brazil is a relatively isolated and socio-ecologically diverse environment, with only limited infrastructure and public services. In the Amazon region, studies have already identified the high use of illicit drugs among adolescents and the high prevalence of HBV and HCV infections among PWUIDs [17, 26, 27]. In this context, this study assessed a sample of 1666 PWUIDs in the Amazon region (Northern Brazil), collecting relevant epidemiological information on HCV infection and related risk factors and outcomes towards improved understanding of HCV transmission pathways and intervention needs in this particular risk population and socio-geographic context.

## Methods

### Study sample and setting

**Fig. 1:**
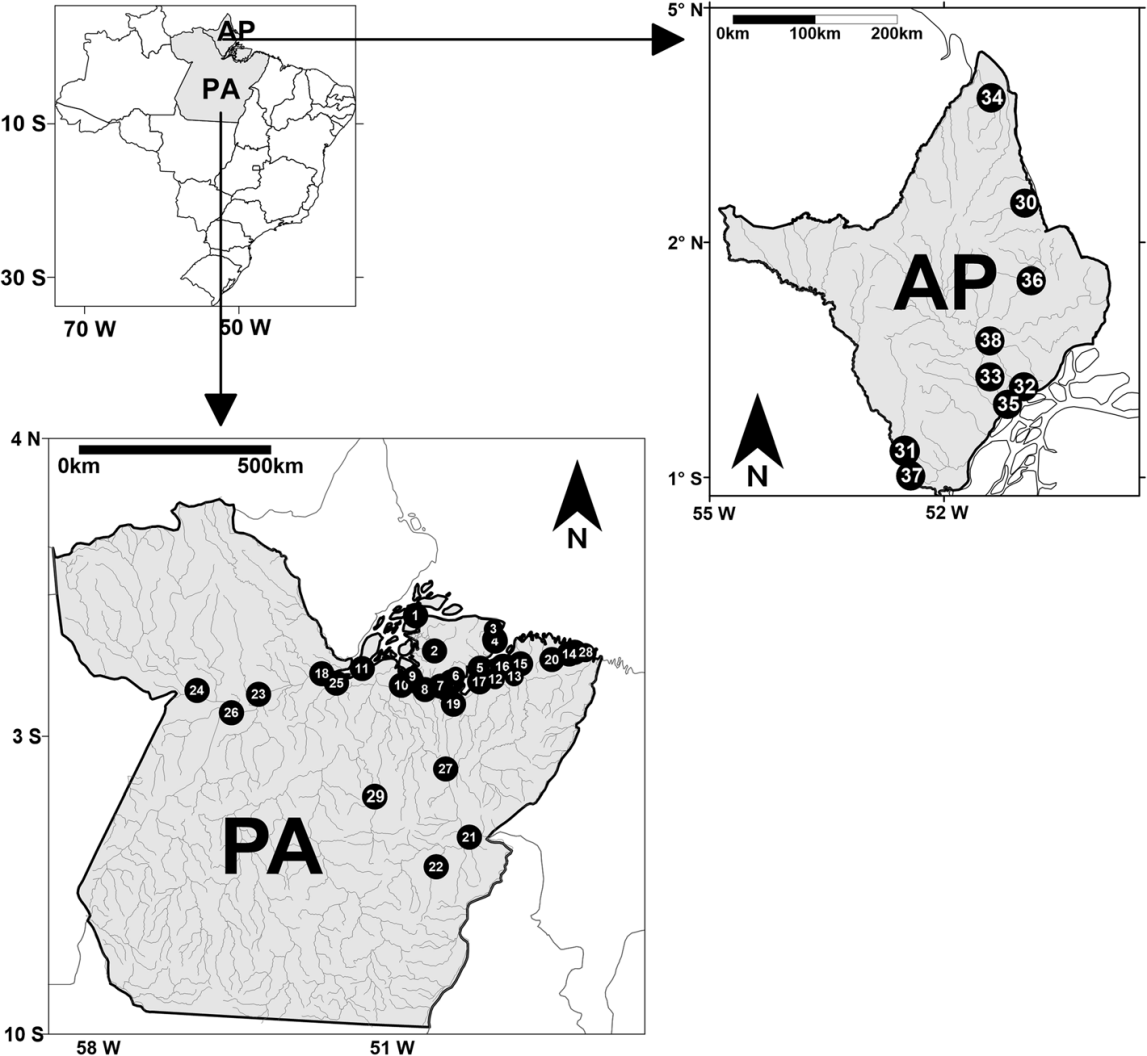
Geographical location of municipalities in the Amazon region that people who used illicit drugs (PWUIDs) were accessed. The Brazilian states of Amapá and Pará are represented by the abbreviations AP and PA, respectively. The numbers from 1 to 38 indicate the municipalities where blood samples were collected and information was provided by the PWUIDs (more details on Additional file 1: Figure S1)

This cross-sectional study relied on biological and self-reported socio-behavioral data from a convenience sample of PWUIDs from the municipalities of the state of Amapá and Pará, in the Amazon region (Northern Brazil) (Fig. 1). Sample recruitment occurred by way of snowball technique^16^. In neighborhoods of the study sites known to be areas of intensive drug consumption, community leaders and other potential recruitment facilitators were identified and invited to assist with participant recruitment for the study. These study facilitators disseminated general information about the study and its objectives in their respective communities, and identified and invited PWUIDs and their relatives and friends as potential study participants. Study eligibility criteria were: (1) use of illicit drugs in the last three months; (2) 18 years of age or older, (3) not under the effect of psychotropic drugs during the meeting with some members of the study team, (4) did not present a risk of death to researchers in the municipality, and (5) willingness to comply with the study protocol, i.e. to provide a biological sample, to complete the epidemiological assessment, and to provide informed consent for study participation.

In each municipality, meetings between researchers and facilitators were held in order to establish the approximate number of PWUIDs to be recruited in each neighborhood, and the local recruitment and assessment procedures to perform the data collection. Potential study candidates were screened for study eligibility (based on the study’s inclusion criteria) during specific field research times in each of the research settings; this procedure was repeated on several occasions. If eligible and consented for the study, participants underwent the study assessment procedures in a private, confidential setting. Several spaces were used to hold meetings with PWUIDs, such as: private rooms in commercial establishments, rooms part of the Universidade Federal do Pará (UFPA) and Universidade Federal do Amapá (UNIFAP), public health facilities, private rooms within schools, churches, and community centers. Members of the research team conducted the interview-based assessments and blood collection of the PWUIDs. All sample and data collections, as well as the formal consents for study participation, were provided on the specific day for each neighborhood of the different municipalities. No PWUIDs received financial incentive to participate in this study. All samples and data were collected between March 2013 and January 2018.

### Data collection

All study participants completed an interviewer-administered questionnaire consisting of question items on socio-demographic, drug use and select clinical/health and behavior outcome variables used in previous studies with similar target populations. The questionnaire contained question items in regards to the following variables: sex, age, colour/race, ethnic origin, marital status, education, amounts and sources of income (last 12 months), blood transfusion and surgical history, tattoos, history and frequency of illicit and other drug use (last 12 months), injection drug use (last 24 months), sharing of drug use equipment (last 12 months), presence of oral or nasal mucosal sores (last 12 months), sexual risk practices (last 12 months), presence of genital ulcers (last 12 months), police arrest/detention (last 12 months), involvement in illicit drug trafficking (last 12 months), and sex work involvement (last 12 months) [16, 24]. In this study, unprotected sexual intercourse and multiple sexual partners were considered sexual risk practices.

### Laboratory tests

Venous blood samples were taken from participants and tested for HCV infection. Specifically, a small blood sample (5 ml) was collected in sterile tubes, allowed to clot, and centrifuged at room temperature. The resulting plasma samples were stored at a temperature below -10 °C. At the end of the collection period at each study site, the samples were transported to UFPA and UNIFAP virological testing laboratories located in Belém, Bragança, Breves and Macapá for laboratory testing. The samples collected were tested for the presence of anti-HCV antibodies and ribonucleic Acid – HCV (HCV-RNA). Anti-HCV antibody was detected using enzyme immunoassay (EIA – Murex anti-HCV 4.0, DiaSorin). The samples were also submitted for the isolation of RNA (QIAmp Viral RNA Mini extraction kit, Qiagen) and transcription of RNA to complementary DNA (cDNA) (High-Capacity cDNA Reverse Transcription kit, Applied Biosystems). These two experiments were performed employing commercial kits according to the manufacturers’ instructions. The 5′ untranslated region (UTR) fragment of HCV was detected by real-time polymerase chain reaction (PCR) [17]. When samples were found to be reactive for anti-HCV but negative for HCV-RNA, further confirmation was done using Immuno Blot Assay (RIBA, Chiron). PWUIDs were categorized as actively infected with HCV if they had a positive HCV-RNA and a positive antibody-HCV test. PWUIDs with non-active (past) HCV infection – or spontaneous clearance - were identified when they had a positive antibody-HCV test and a negative HCV-RNA test. The samples presenting HCV-RNA had their genotypes identified by real-time PCR [17].

### Data processing and statistical analysis

All study data collected - including epidemiological indicator data from the participants’ questionnaires - were entered into an Excel database and converted to SPSS. The proportion of participants who had active HCV and non-active (past) HCV infection was assessed. Confidence intervals were computed to estimate the prevalence of HCV infection rates. A descriptive analysis was conducted to investigate the bivariate relationships between HCV status and epidemiological (i.e., socio-demographic, drug use and other health-related) covariates drawn from the epidemiological questionnaire data. All the potential factors with probabilities of *p* ≤ 0.2 were examined and included in the final models of HCV infection (i.e. active + non-active infections) and HCV spontaneous clearance (HSC) status, using backward stepwise multiple logistic regression. Multiple logistic regressions were then run to determine the association of each factor with HCV infection and HSC status. Various possible types of interactions were evaluated in order to determine how they might improve the final models [28]. The fit of the final model was assessed using the Hosmer-Lemeshow goodness-of-fit test. All statistical analyses were performed using SPSS 20.0 for Windows, with a level of significance of 0.05.

## Results

### Characteristics of PWUIDs

Of the total sample of 1666 participants recruited into the study, 539 PWUIDs were from nine municipalities of the state of Amapá (32.4%) and 1127 PWUIDs were from twenty-nine municipalities of the state of Pará (67.6%) (Fig. 1). The number of PWUIDs accessed in each municipality is available in the supporting information (Additional file 1: Figure S1). Respective majorities of the PWUIDs sample were male, aged between 18 to 29 years, heterosexual, single, Brazilian and born in the Amazon region, non-white and with a low/limited level of education. Most participants reported their initial onset of illicit drug use during adolescence, i.e. below the age of 18 years (64.9%). The average monthly income of PWUIDs was around the Brazilian minimum wage standard (approximately 210 US dollars/month), derived from formal or informal employment sources (Table 1).

**Table 1:** Socio-demographic and economic characteristics of the sample of people who used illicit drugs related to the status of HCV infection

Characteristics	N	HCV infection		
		Active % (n)	Non-active % (n)	Total % (n)
Total sample	1666	23.0 (384)	11.6 (193)	34.6 (577)
Sex				
Male	1053	23.8 (251)	10.5 (111)	34.3 (362)
Female	600	22.0 (132)	13.7 (82)	35.7 (214)
Transgendered	13	7.7 (1)	0.0 (0)	7.7 (1)
Age				
18–29 years	893	21.1 (188)	13.1 (117)	34.2 (305)
30–39 years	575	21.0 (121)	8.9 (51)	29.9 (172)
40 + years	198	37.9 (75)	12.6 (25)	50.5 (100)
Origin				
Brazilian – Born in the Amazon region	1426	21.0 (299)	13.1 (187)	34.1 (486)
Brazilian – Not born in the Amazon region	224	37.0 (83)	2.7 (6)	39.7 (89)
Non-Brazilian	162	1.2 (2)	0.0 (0)	1.2 (2)
Colour/race				
White	564	27.3 (154)	9.2 (52)	36.5 (206)
Non-white	1102	20.9 (230)	12.8 (141)	33.7 (371)
Sexual orientation				
Heterosexual	1507	23.0 (346)	11.8 (178)	34.8 (524)
Same-sex (including bisexual)	159	23.9 (38)	9.4 (15)	33.3 (53)
Marital status^†^				
Single, separated or widowed	1036	23.4 (242)	10.9 (113)	34.3 (355)
Married or co-habitating	630	22.5 (142)	12.7 (80)	35.2 (222)
Education				
No formal education/some elementary school	1009	23.0 (232)	10.8 (109)	33.8 (341)
Completed elementary school or higher	657	23.1 (152)	12.8 (84)	35.9 (236)
Monthly income^†^				
Less than Brazilian minimum wage	1118	21.5 (241)	11.9 (133)	33.4 (374)
More than Brazilian minimum wage	548	26.1 (143)	10.9 (60)	37.0 (203)
Income source^†^				
Regular or irregular job	1039	21.4 (222)	9.7 (101)	31.1 (323)
Social benefits/pension	221	14.0 (31)	17.2 (38)	31.2 (69)
Criminal activity	406	32.3 (131)	13.3 (54)	45.6 (185)

^†^Last 12 months

All PWUIDs reported frequent use of non-injected drugs in the last 12 months. A sub-group of PWUIDs reported injecting drug use (cocaine) at least once (last 24 months) (14.9%). Most PWUIDs had used illicit drugs for a period of between 5 to 15 years (60.3%), with an average period of 7.5 years. Crack-cocaine or related products (oxi) was the main drug used by most participants (50.2%) (Table 2). Most PWUIDs reported using more than one illicit drug during their lifetime (92.2%), commonly involving marijuana as the initial illicit drug used. The sharing of drug use equipment was common among PWUIDs (*n* = 988, 59.3%) (Table 2), specifically inhalation-pipe sharing was reported by many users of non-injecting drugs (*n* = 795, 47.7%), and syringe and needle sharing among injecting drug users (*n* = 193, 11.6%). Some crack-cocaine users reported sores in their oral or nasal mucosa (34.5%). Participation in the distribution or sale of illicit drugs (17.0%) and a history of police or in prison detention (23.4%) was reported by some PWUIDs (Table 2). Many users reported risky sexual practice or compromised sexual health, including multiple sexual partners and sex work involvement (34.8%), and genital ulcers (46.9%).

**Table 2:** Sample characteristics related to illicit drug use, illicit activities, sex and risks associated with the status of HCV infection

Characteristics	N	HCV infection		
		Active % (n)	Non-active % (n)	Total % (n)
Total sample	1666	23.05 (384)	11.58 (193)	34.63 (577)
Main illicit drug used^a^				
Marijuana	137	9.5 (13)	4.4 (6)	13.9 (19)
Crack/oxi	837	25.6 (214)	11.7 (98)	37.3 (312)
Cocaine	692	22.7 (157)	12.9 (89)	35.6 (246)
Daily use of illicit drugs^a^	1153	29.0 (335)	13.8 (159)	42.8 (494)
Over 12 years using illicit drugs	549	56.4 (310)	27.0 (148)	83.4 (458)
Injecting drug use^b^	248	37.5 (93)	17.3 (43)	54.8 (136)
Sharing of drug use equipment^a^	988	31.5 (311)	16.1 (159)	47.6 (470)
Involvement in illicit drug trafficking^a^	283	47.0 (133)	18.4 (52)	65.4 (185)
Detention (by police or in prison) ^a^	373	22.8 (85)	14.7 (55)	37.5 (140)
Unprotected sexual intercourse^a^	1387	25.0 (347)	10.7 (149)	35.7 (496)
More than 12 sexual partners^a^	828	26.0 (215)	11.6 (96)	37.6 (311)
Sex work involvement^a^	663	21.3 (141)	11.9 (79)	33.2 (220)
Blood transfusion history	283	31.1 (88)	6.7 (19)	37.8 (107)
Tattoos	1082	29.4 (318)	11.5 (124)	40.9 (442)

^a^Last 12 months. ^b^Last 24 months

### HCV infection status and distribution of genotypes

In total, 577 PWUIDs (36.6%) had anti-HCV antibodies by EIA. Among these, 384 PWUIDs also featured HCV-cDNA (23.0%), indicating active HCV infection. Conversely, 193 PWUIDs had anti-HCV antibodies by EIA and immune-blot, with absence of HCV-cDNA by real-time PCR (11.6%), indicating non-active HCV infection, and presented with presumed spontaneous clearance of HCV (Tables 1, 2 and 3). In PWUIDs with HCV-cDNA, genotypes 1 (80.2%) and 3 (18.8%) were identified, including four co-infections with these HCV genotypes (1.0%) (Table 3).

**Table 3:** Prevalence of infection and distribution of HCV genotypes among people who used illicit drugs

Marker	Prevalence		95% CI
	Positive/Total	%	
HCV infection			
All (EIA+)	577/1666	34.6	28.3–41.1
Active (EIA+ and PCR+)	384/1666	23.0	17.9–28.9
Non-active (EIA+ Immuno Blot+ and PCR -)	193/1666	11.6	5.5–18.4
Genotypes			
Genotype 1	308/384	80.2	75.7–85.2
Genotype 3	72/384	18.8	12.7–25.0
Genotype 1 + Genotype 3	4/384	1.0	0.0–4.6

### Characteristics associated with HCV infection

The univariate analysis identified the following socio-behavioral characteristics associated with all HCV infection (i.e. active + non-active infections): age ≥ 30 years, use of crack/oxi + cocaine, daily drug use, > 12 years drug use history, injection drug use, sharing of drug use equipment, involvement in illicit drug trafficking, unsafe sex practices, > 12 sexual partners, and a history of tattoos (Table 4). The multivariate analysis identified an association of the same ten characteristics with all HCV infection. The Hosmer-Lemeshow test indicated that the final model (_HL_χ^2^ = 11.23; *p* = 0.25) had an overall good fits. The characteristic most strongly associated with all HCV infection was an illicit drug use history of > 12 years (OR = 27.55). Moreover, two characteristics were associated with non-active HCV infection, or HSC, by way of univariate and multivariate analysis: Brazilian origin (born in the Amazon region) and non-white ethnicity (Table 5). The Hosmer-Lemeshow test indicated that the final model (_HL_χ^2^ = 0.32; *p* = 0.59) had a good fit. Brazilian origin (born in the Amazon region) was the main characteristic associated with HSC (OR = 8.84).

**Table 4:** Bivariate and multivariate analysis of risk factors for HCV infection

Risk factors	All HCV infection	
	Bivariate OR (95% CI)	Multivariate aOR (95% CI)
Age		
Up to 29 years	1.00	1.00
≥ 30 years	1.29 (1.02–1.64)	1.10 (1.01–1.75)
Main illicit drugs used^a^		
Marijuana	1.00	1.00
Crack/oxi + Cocaine	3.58 (2.15–5.87)	3.24 (2.03–5.38)
Frequency of use of illicit drugs^a^		
Non-daily	1.00	1.00
Daily	3.89 (2.96–5.06)	4.01 (2.85–5.62)
Over 12 years using illicit drugs		
No	1.00	1.00
Yes	29.52 (13.47–52.31)	27.55 (13.78–50.23)
Injection drug use^b^		
No	1.00	1.00
Yes	2.71 (2.03–3.57)	3.04 (2.35–4.02)
Sharing of drug use equipment^a^		
No	1.00	1.00
Yes	4.64 (3.80–6.36)	4.18 (3.54–6.07)
Involvement in illicit drug trafficking^a^		
No	1.00	1.00
Yes	4.77 (3.61–7.16)	4.26 (3.35–7.49)
Unprotected sexual intercourse^a^		
No	1.00	1.00
Yes	1.37 (1.02–1.81)	1.22 (1.01–1.69)
More than 12 sexual partners^a^		
No	1.00	1.00
Yes	1.28 (1.05–1.59)	1.19 (1.02–1.75)
Tattoos		
No	1.00	1.00
Yes	4.14 (3.11–5.32)	3.96 (2.84–4.95)

^a^Last 12 months. ^b^Last 24 months. Factors not associated with HCV infection can be accessed in Additional file 1: Table S1. OR: Odds Ratio. aOR: adjusted Odds Ratio. 95% CI: 95% Confidence interval

**Table 5:** Bivariate and multivariate analysis of factors associated with HCV spontaneous clearance

Factors	HCV spontaneous clearance	
	Bivariate OR (95% CI)	Multivariate aOR (95% CI)
Origin		
Brazilian + Non-Brazilian – Not born in the Amazon region	1.00	1.00
Brazilian – Born in the Amazon region	8.84 (3.75–19.81)	7.43 (2.54–18.72)
Colour/race		
White	1.00	1.00
Non-White	1.80 (1.22–2.71)	2.02 (1.28–3.11)

Factors not associated with HCV spontaneous clearance can be accessed in Additional file 1: Table S1. *OR* Odds Ratio, *aOR* Adjusted Odds Ratio, *95% CI* 95% Confidence interval

## Discussion

This study identified important characteristics of the study population of PWUIDs in the Amazon region of Northern Brazil and, specifically, associations with HCV infection status in this vulnerable group. The sample was predominantly composed of men, non-white, young, poor, unmarried, heterosexual and limited education levels. Moreover, some PWUIDs have indicated involvement with sex work, illicit drug trafficking, and the criminal justice system. This information is consistent with findings from other studies in Brazil [13, 18, 21, 29, 30]. Some studies have indicated that socioeconomic marginalization contributes to an increased risk of morbidity and mortality among PWUIDs [30, 31]. The strong association between HCV infection and involvement with drug trafficking may represent an example of socioeconomic marginalization contributing to increase the health risks of the PWUIDs. The role of these in the specific study sample requires further examination, which should be done in the future.

Crack and cocaine (used mainly non-injection) were the main illicit drugs consumed by study participants. However, many users may be considered poly-drug users, including some of them reporting - occasional - injection drug use at some point in life. Daily drug use and sharing of drug use equipment were characteristics common for most of the study participants. These findings corroborate information provided in other epidemiological studies conducted with PWUIDs in Brazil, including from the Amazon region [16, 17, 19–21, 24–27, 29, 30].

The estimated prevalence of HCV infection in the Amazon region’s general population ranges from 1 to 3%, based on the diagnostic methods used, with higher rates being recorded in different sub- or risk groups (e.g., older ages, indigenous) [22, 32–34]. HCV prevalence estimates from previous studies have been multifold higher among PWUIDs, ranging from 28 to 37%, compared to general populations [16, 17, 23–25]. The present study confirmed the high prevalence of HCV infection (36.6%) among PWUIDs in the Amazon region, reinforcing the need for effective control and prevention measures. The predominance of genotype 1 was observed among PWUIDs, but frequency of the genotype 3 was elevated relative to other risk population in the Amazon region, such as patients with chronic hematologic diseases (recipients of multiple blood transfusions) and patients undergoing hemodialysis [35, 36]. Most HCV infections in the Amazon region consist of genotype 1, whereas genotype 3 is found mostly among PWUIDs [16, 17, 24, 32, 35–37]. The rapid spread of genotypes 1 and 3 (and specifically subtypes 1a and 3a), in different geographic areas over the past decades, has been a consequence of efficient transmission through unsafe blood transfusion products and injecting drug use [38–40].

This study is a first in describing HCV infection status (i.e. active and non-active) in a multi-site PWUIDs population in the Amazon region in Northern Brazil. Among 577 PWUIDs infected with HCV, 193 (33.4%) presented with HCV spontaneous clearance. The multivariate analysis suggested spontaneous clearance status to be strongly associated with origin (Amazonian-born) and non-white (e.g., Black or Indigenous) of the participants. The contemporary population of Northern Brazil is comprised of a heterogeneous socio-ethnic mixture of indigenous Brazilian natives combined with migratory populations of Caucasians and Blacks of diverse origins [41]. Likely, a predominance of indigenous descent, and associated genetic factors, is a variable behind this strong association of socio-ethnic origin with HCV spontaneous clearance. Genetic ancestry analysis markers were not used in this study, but related finding have also been detected in other studies, including native/indigenous people in North America [5, 42]. In Canada, a study conducted with PWUIDs found that Indigenous/Aboriginal status was a factor associated with HCV spontaneous clearance [42]. The understanding of factors that promote or prevent the generation of protective immunity is potentially crucial for the development especially of improved prevention (e.g., a possible HCV vaccine) and/or treatment tools for HCV infection; further studies are needed from a clinical and virological perspective on these issues.

HCV infection status was associated with several risk factors in this study, most of which have previously been identified by other studies in Brazil [17, 19–21, 24, 43]. The presence of tattoos and older age (> 30 years) are characteristic known to be associated with HCV infection in PWUIDs and Amazonian blood donors [16, 17, 24, 25, 28, 32]. Longer histories (> 12 years) of illicit drug use was the characteristic most strongly associated with HCV infection status, implying extended opportunity for and exposure to repeated key risk behaviors facilitating the acquisition of HCV and other pathogens, such as: sharing of drug use equipment, and injection drug use [16, 17, 19–21]. The active involvement with drug trafficking as a risk factor likely provides quick and easy access to a greater number of different drugs (e.g., injectable cocaine) as well as riskier use methods (e.g., drug injecting), and so provides increased risk pathways to HCV transmission. Moreover, those newly infected with HCV can continue to transmit HCV to other users through the shared use of non-injection equipment (i.e., crack smoking paraphernalia). The possibility of HCV transmission by shared non-injection equipment has been demonstrated by studies in several countries, including studies in the Amazon region [24, 43–46]. Recently, the presence of HCV-RNA in paraphernalia for crack-cocaine consumption (pipes and aluminum cans) was detected among samples from the Amazon region [46]. Studies have suggested that the risk of HCV transmission by shared non-injection paraphernalia would be modulated by the presence oral wounds and types of paraphernalia (i.e., sharp or heat-intensive materials) used [44–46].

Sexual transmission of HCV is still controversially discussed, but considered possible [47]. Sexual transmission of HCV has been more commonly recorded in men-who-have-sex-with-men featuring high sexual (e.g., HIV) and drug use risks status [48]. Other studies have indicated the possibility of sexual transmission of HCV among PWUIDs with sexually transmitted infections (STIs), especially with the presence of oral and/or genital wounds [49, 50]. High levels of syphilis and HCV-HIV co-infection among PWUIDs have been documented previously in the Amazon region [17, 24, 29, 51]. These dynamics likely are involved in the associations of unprotected sexual intercourse with HCV infection status in the present study sample. An evaluation of HCV co-infections with other pathogens such as HBV, HIV, HTLV and *T. pallidum* in the present sample – confounded by poor health care resources and infrastructure in the study region – will be carried out separately to further examine this comprehensive epidemiological-virological picture.

The present study has a number of possible limitations. One limiting factor was the age limit criterion (18 years) applied for eligible participants, since many PWUIDs report an onset of illicit drug use at ages < 18 years. Second, the restriction of the study to the municipalities of the Amazon region renders the study sample not necessarily representative of the PWUIDs population in Northern Brazil more generally. In addition, although snowball sampling has been found to be adequate for quasi-representative sampling in hidden populations, could have been used to improve representativeness. As the interview data are self-reported, some information, such as drug use or sex-related risks behaviors, may contain response or recall bias. Screening for HCV infection was based on EIA, recent infections may present a small concentration of anti-HCV antibodies, not yet detected by EIA, and therefore may have been diagnosed as negative. Finally, the cross-sectional design of the study limits its capacity to establish causality.

## Conclusions

The present study is the first epidemiological examination of HCV infection (active or non-active) status and related risk factors among a large, multi-site sample of PWUIDs in the Amazon region. High levels of HCV infection – predominantly genotype 1 and associated drug use intensity, sexual risks and social marginalization – as well as spontaneous clearance were identified. The results, despite the context of an underdeveloped health care structure, emphasize the urgent need for improved HCV prevention and care services for marginalized drug users in the Brazilian Amazon region, yet also for improved understanding of HCV transmission risks and especially those factors facilitating spontaneous clearance for HCV in the study’s target population.

## Additional file

Additional file 1: **Figure S1.** Sample of people who used illicit drugs accessed in each municipality in this study. **Table S1.** Univariate and multivariate analysis of factors not associated with HCV infection and HCV spontaneous clearance. (DOCX 49 kb)

## Data Availability

The datasets analyzed during the current study are not publicly available due to the progress of analyzes of possible infections and co-infections with other pathogens, but are available from the corresponding author on reasonable request.

## Abbreviations

95% CI: 95% Confidence interval
aOR: Adjusted Odds Ratio
cDNA: Complementary DNA
EIA: Enzyme immunoassay
HCV: Hepatitis C virus
HSC: HCV spontaneous clearance
OR: Odds Ratio
PCR: Polymerase chain reaction
PWUIDs: People who used illicit drugs
RIBA: Immuno Blot Assay
RNA: Ribonucleic Acid
UFPA: Universidade Federal do Pará
UNIFAP: Universidade Federal do Amapá
UTR: Untranslated region
