# Supplementary material for: Hepatitis C virus infection status and associated factors among a multi-site sample of people who used illicit drugs in the Amazon region
Source: BMC Infect Dis. 2019 Jul 17;19:634. doi: 10.1186/s12879-019-4270-2 (PMC6637600; doi:10.1186/s12879-019-4270-2)
Supplement: Supplementary file 1 — Figure S1. Sample of people who used illicit drugs accessed in each municipality in this study. Table S1. Univariate and multivariate analysis of factors not associated with HCV infection and HCV spontaneous clearance. (DOCX 49 kb) [file 12879_2019_4270_MOESM1_ESM.docx]

Additional file 1

**Hepatitis C virus infection status and associated factors among a multi-site sample of people who used illicit drugs in the Amazon Region.**

Figure S1: Sample of people who used illicit drugs (PWUIDs) accessed in each municipality.

| Municipality (Brazilian state) | Number in figure 1 | Number of PWUIDs accessed |
| --- | --- | --- |
| Afuá (Pará) | 1 | 21 |
| Anajás (Pará) | 2 | 22 |
| Soure (Pará) | 3 | 27 |
| Salvaterra (Pará) | 4 | 23 |
| Ponta de Pedras (Pará) | 5 | 18 |
| São Sebastião da Boa Vista (Pará) | 6 | 19 |
| Curralinho (Pará) | 7 | 85 |
| Bagre (Pará) | 8 | 25 |
| Breves (Pará) | 9 | 187 |
| Melgaço (Pará) | 10 | 22 |
| Gurupá (Pará) | 11 | 17 |
| Belém (Pará) | 12 | 105 |
| Benevides (Pará) | 13 | 21 |
| Bragança (Pará) | 14 | 55 |
| Castanhal (Pará) | 15 | 34 |
| Marituba (Pará) | 16 | 68 |
| Abaetetuba (Pará) | 17 | 36 |
| Almeirim (Pará) | 18 | 11 |
| Cametá (Pará) | 19 | 17 |
| Capanema (Pará) | 20 | 36 |
| Marabá (Pará) | 21 | 52 |
| Parauapebas (Pará) | 22 | 35 |
| Monte Alegre (Pará) | 23 | 18 |
| Óbidos (Pará) | 24 | 12 |
| Porto de Moz (Pará) | 25 | 15 |
| Santarém (Pará) | 26 | 59 |
| Tucuruí (Pará) | 27 | 38 |
| Augusto Correa (Pará) | 28 | 27 |
| São Felix do Xingu (Pará) | 29 | 22 |
| Calçoene (Amapá) | 30 | 47 |
| Laranjal do Jari (Amapá) | 31 | 49 |
| Macapá (Amapá) | 32 | 119 |
| Mazagão (Amapá) | 33 | 58 |
| Oiapoque (Amapá) | 34 | 57 |
| Porto Grande (Amapá) | 35 | 38 |
| Santana (Amapá) | 36 | 100 |
| Tartarugalzinho (Amapá) | 37 | 43 |
| Vitória do Jari (Amapá) | 38 | 28 |

Table S1: Bivariate and multivariate analysis of factors not associated with HCV infection and HCV spontaneous clearance.

| Risk factors |  | All HCV infection | |  | HCV Clearence | |
| --- | --- | --- | --- | --- | --- | --- |
|  |  | Bivariate OR | Multivariate aOR |  | Bivariate OR | Multivariate aOR |
| Sex |  |  |  |  |  |  |
| Female |  | 1.00 |  |  | 1.00 |  |
| Male |  | 0.95 (0.75 - 1.17) |  |  | 0.72 (0.48 - 1.03) |  |
| Age |  |  |  |  |  |  |
| Up to 29 years |  |  |  |  | 1.00 |  |
| ≥ 30 years |  |  |  |  | 1.05 (0.71 - 1.52) |  |
| Source |  |  |  |  |  |  |
| Brazilian + Non-Brazilian – Not born in the Amazon |  | 1.00 |  |  |  |  |
| Brazilian – Born in the Amazon |  | 0.79 (0.57 - 1.05) |  |  |  |  |
| Colour/race |  |  |  |  |  |  |
| White |  | 1.00 |  |  |  |  |
| Non-White |  | 0.89 (0.70 - 1.10) |  |  |  |  |
| Sexual orientation |  |  |  |  |  |  |
| Heterosexual |  | 1.00 |  |  | 1.00 |  |
| Homosexual + Bisexual |  | 0.92 (0.64 - 1.34) |  |  | 0.75 (0.40 - 1.44) |  |
| Marital status* |  |  |  |  |  |  |
| Single or separated |  | 1.00 |  |  | 1.00 |  |
| Married or co-habitating |  | 1.03 (0.83 - 1.31) |  |  | 1.21 (0.83 - 1.73) |  |
| Education |  |  |  |  |  |  |
| Completed elementary school or higher |  | 1.00 |  |  | 1.00 |  |
| No formal education/some elementary school |  | 0.91 (0.72 - 1.13) |  |  | 0.85 (0.58 - 1.22) |  |
| Monthly income* |  |  |  |  |  |  |
| More than one Brazilian minimum wage |  | 1.00 |  |  | 1.00 |  |
| Up to one Brazilian minimum wage |  | 0.84 (0.68 - 1.07) |  |  | 0.92 (1.29 - 1.92) |  |
| Source of income* |  |  |  |  |  |  |
| Regular or irregular job + Social benefits/pension |  | 1.00 |  |  | 1.00 |  |
| Criminal activity |  | 1.21 (0.91 - 1.64) |  |  | 0.76 (0.50 - 1.10) |  |
| Main illicit drugs used* |  |  |  |  |  |  |
| Marijuana |  |  |  |  | 1.00 |  |
| Crack/oxi + Cocaine |  |  |  |  | 1.10 (0.39 - 2.93) |  |
| Frequency of use of illicit drugs* |  |  |  |  |  |  |
| Non-daily |  |  |  |  | 1.00 |  |
| Daily |  |  |  |  | 0.67 (0.41 - 1.12) |  |
| Over 12 years using illicit drugs |  |  |  |  |  |  |
| No |  |  |  |  | 1.00 |  |
| Yes |  |  |  |  | 0.77 (0.52 - 1.23) |  |
| Injection drug use** |  |  |  |  |  |  |
| No |  |  |  |  | 1.00 |  |
| Yes |  |  |  |  | 0.91 (0.57 - 1.38) |  |
| Sharing of drug use equipment* |  |  |  |  |  |  |
| No |  |  |  |  | 1.00 |  |
| Yes |  |  |  |  | 1.10 (0.69 - 1.73) |  |
| Involvement in illicit drug trafficking* |  |  |  |  |  |  |
| No |  |  |  |  | 1.00 |  |
| Yes |  |  |  |  | 0.70 (0.48 - 1.03) |  |
| Detention (by police or in prison)* |  |  |  |  |  |  |
| No |  | 1.00 |  |  | 1.00 |  |
| Yes |  | 1.18 (0.91 - 1.51) |  |  | 1.40 (0.93 - 2.09) |  |
| Unsafe sex practices* |  |  |  |  |  |  |
| No |  |  |  |  | 1.00 |  |
| Yes |  |  |  |  | 0.64 (0.39 - 1.06) |  |
| More than 12 sexual partners* |  |  |  |  |  |  |
| No |  |  |  |  | 1.00 |  |
| Yes |  |  |  |  | 0.77 (0.54 - 1.13) |  |
| Involvement in prostitution* |  |  |  |  |  |  |
| No |  | 1.00 |  |  | 1.00 |  |
| Yes |  | 0.88 (0.71 - 1.12) |  |  | 1.18 (0.81 - 1.72) |  |
| Blood transfusion history |  |  |  |  |  |  |
| No |  | 1.00 |  |  | 1.00 |  |
| Yes |  | 1.16 (0.89 - 1.55) |  |  | 0.68 (0.42 - 1.12) |  |
| Tattoos |  |  |  |  |  |  |
| No |  |  |  |  | 1.00 |  |
| Yes |  |  |  |  | 0.67 (0.40 - 1.07) |  |
